# Pathogen Security-Help or Hindrance?

**DOI:** 10.3389/fbioe.2014.00083

**Published:** 2015-01-06

**Authors:** Stephen A. Morse

**Affiliations:** ^1^Centers for Disease Control and Prevention, Atlanta, GA, USA

**Keywords:** pathogen security, select agents, biosecurity, biosafety, bioterrorism

## Abstract

Events over the past 15 years have resulted in the promulgation of regulations in the United States to enhance biosecurity by restricting the access to pathogens and toxins (i.e., biological select agents and toxins [BSATs]), which pose a severe threat to human being, animal, or plant health or to animal or plant products, to qualified institutions, laboratories, and scientists. These regulations also reduce biosafety concerns by imposing specific requirements on laboratories working with BSATs. Furthermore, they provide a legal framework for prosecuting someone who possesses a BSAT illegally. With the implementation of these regulations has come discussion in the scientific community about the potential of these regulations to affect the cost of doing BSAT research, hamper research and international collaborations, or whether it would stop someone with a microbiological background from isolating many of the select agents from nature.

“Biosafety and biosecurity measures contribute to preventing the development, acquisition or use of biological and toxin weapons….”

–(BWC Meeting of States Parties, 2008)

## Introduction

Recent events have changed the manner in which scientists acquire and work with pathogenic microorganisms and biological toxins (Morse and Weirich, 2011). Changes in how scientists conduct research on biological select agents and toxins (BSATs) have occurred not only in the United States (U.S.) but internationally as well. It has been influenced by a number of terrorist events, which have increased national and international awareness of the threat of terrorism (including bioterrorism) such as the unsuccessful anthrax attack in Kameido, Tokyo in 1993 (Kaplan, 2000; Takahashi et al., 2004; Danzig et al., 2011) and the release of the nerve agent sarin in the subway system of Tokyo in 1995 by the Aum Shinrikyo (Olsen, 1999; Kaplan, 2000; Danzig et al., 2011); the bombing of the Murrah Federal Building in Oklahoma City in 1995 (Smoak and Geiling, 2004); the terrorist attacks using commercial airlines on September 11, 2001 (Smoak and Geiling, 2004); and the 2001 anthrax attacks in the U.S. (Cole, 2003). In an effort to control the misuse of these dangerous pathogens and toxins by those wishing to do harm, the U.S. passed legislation and promulgated regulations designed to limit unauthorized access to these agents. Following the implementation of these regulations, Casadevall and Relman (2010) proposed that the policies may have the unintended consequence of hindering necessary research on countermeasures and potentially increasing societal vulnerability to biological attacks and natural epidemics. The international community has responded in a variety of ways to the threat of bioterrorism, which provides additional perspectives on the impact of policy solutions in this area. For example, several countries have developed and implemented laboratory biosecurity legislation to regulate possession, use and access to biological agents while other countries use existing biosafety legislation for pathogen security. This article will cover four areas: (1) a review of the events that led up to the development of regulations to restrict access to dangerous pathogens in the U.S.; (2) a discussion of the laws that framed the regulations; (3) the positive and negative effects these regulations have had on the scientific community; and (4) policy options provided by international approaches for pathogen security.

## Definitions

For the purposes of this paper, the following definitions will be used:

The term *pathogen security* refers to “measures to reduce the risk of bioterrorism by making it harder for would-be perpetrators to gain access to dangerous pathogens and toxins that have legitimate uses in biomedical research but could be misused for the development of biological weapons” (Tucker, 2007). Laboratory *biosafety* is defined as “the containment principles, technologies, and practices that are implemented to prevent the unintentional exposure to pathogens and toxins, or their accidental release” (World Health Organization, 2004). *Biosecurity* is defined as “a set of institutional and personal security measures designed to prevent the loss, theft, misuse, diversion, or intentional release of biological materials that could be used with intent to harm people, livestock, agriculture, or the environment” (World Health Organization, 2004, 2006). *Risk*, as it is used to identify agents that may be used for bioterrorism, is defined as “the product of probability and impact of an adverse event” (International Organization for Standardization, 2009). *Biological surety* (or *biosurety*) is primarily a U.S. Department of Defense (DoD) program to “safeguard BSAT from theft and misuse while ensuring that BSAT work is conducted safely” (Department of the Army, 2008). *Dual-use research of concern* has been defined by the National Science Advisory Board for Biosecurity (NSABB) “as a research that, based on current understanding, can be reasonably anticipated to provide knowledge, products, or technologies that could be directly misapplied by others to pose a threat to public health and safety, agricultural crops and other plants, animals, the environment, or materiel” (National Security Advisory Board for Biosecurity, 2007).

## Historical Perspective

The events leading up to the promulgation of regulations restricting access to dangerous human pathogens and biological toxins began in May 1995 when Larry Wayne Harris, a resident of Lancaster, Ohio with microbiology training and ties to the white supremacist group Aryan Nations, ordered three vials of lyophilized *Yersinia pestis* from the American Type Culture Collection (ATCC) (Stern, 2000; Carus, 2002). At the time, no law prohibited Mr. Harris or anyone from acquiring this dangerous pathogen, even though it killed a quarter of Europe’s population in the mid-fourteenth century (Stern, 2000). Mr. Harris was informed by the ATCC that he had to run an established laboratory in order to set up an account and place orders. He faxed fraudulent information on falsified letterhead stationary that mislead the ATCC to believe that the address, which was actually his home, was that of a legitimate laboratory. Once the account was approved, he phoned the ATCC and ordered three vials of *Y. pestis*, which were shipped 4 days later. In the meantime, Mr. Harris had become impatient and called the ATCC to complain that he had not received the shipment. ATCC personnel became suspicious when he indicated the nature of his planned experiments and notified the Centers for Disease Control and Prevention (CDC). CDC spoke with Mr. Harris and upon ascertaining that the laboratory was in his home notified the Ohio Department of Health, which in turn notified the Lancaster Health Department. The Lancaster Health Department then notified local law enforcement authorities. Subsequently, three unopened, intact vials containing *Y. pestis* were retrieved from the glove compartment of Mr. Harris’s car by local law enforcement authorities using a search warrant. Mr. Harris eventually pled guilty to one count of wire fraud.

Shortly after this highly publicized incident, a review of Federal regulations governing the possession of dangerous pathogens, initiated by the National Security Council, identified several regulations that restricted the possession, transfer, and use of high consequence plant and animal pathogens to qualified institutions, laboratories, and scientists. However, similar regulations that would restrict access to pathogens, toxins, and recombinant organisms dangerous to human beings were not found. A multi-agency committee was commissioned by the Secretary of the Department of Health and Human Services (HHS) to address this issue and close the loophole. The committee, co-chaired by Frank Young of the Office of Emergency Preparedness, HHS and Stephen A. Morse of CDC, HHS included representatives from the Office of Science and Technology Policy, Executive Office of the President; the Federal Bureau of Investigation (FBI); Department of Justice (DOJ); Department of Defense [U.S. Army Medical Research Institute for Infectious Diseases (USAMRIID)]; Department of Agriculture (USDA); Department of Commerce; Environmental Protection Agency; U. S. Postal Service; National Institutes of Health; Food and Drug Administration; Office of Emergency Preparedness; and CDC (Tipple et al., 1997). A framework for a solution was developed by the committee and presented on March 6, 1996, during CDC testimony at a hearing of the Senate Judiciary Committee convened to examine concerns arising from the interstate transportation of human pathogens (Tipple et al., 1997). This framework was subsequently incorporated into the Antiterrorism and Effective Death Penalty Act of 1996 (Public Law 104-132), which was signed into law on April 24, 1996. Section 511 of this law directed the HHS Secretary (and ultimately CDC) to promulgate regulations to establish and maintain a list of biological agents that have the potential to pose a severe threat to public health and safety. This list subsequently became known as the Select Agent List. In determining whether to include an agent on this list, the committee was instructed to use the following criteria: (1) the effect on human health from infection by the agent; (2) the degree of contagiousness of the agent and the route(s) of infection; and (3) the availability and effectiveness of vaccines to prevent disease and medical countermeasures or therapeutics to treat any illness resulting from infection caused by the agent (Public Law 104-132, 1996).

On March 12, 1996, CDC representatives attended a meeting of the Biological Weapons Task Force of the American Society for Microbiology (ASM), which was chaired by Kenneth I. Berns. The purpose of this meeting was to discuss the framework that was presented during CDC testimony at the March 6 meeting of the Senate Judiciary Committee and its subsequent implementation.

Further input from the scientific community was sought after the Notice of Proposed Rulemaking was published in the *Federal Register* on June 10, 1996 (Department of Health and Human Services, 1996a). The ASM assisted in this endeavor by contacting more than 11,000 of its members requesting comments. Pertinent comments and suggestions that were received from ASM members as well as from other scientists were addressed in the Preamble to the Final Rule, and many were incorporated into the Final Rule (Department of Health and Human Services, 1996b). The new regulation required those shipping or receiving (i.e., transfer) BSAT to register with the CDC. The regulation also required that safety procedures for agent transfer be established and enforced, that those handling these agents be properly trained, and that there are proper laboratory facilities to contain and dispose of the agents. In order to accomplish this, the CDC/NIH publication “Biosafety in Microbiological and Biomedical Laboratories (BMBL), 4th Edition” was incorporated by reference into the regulation. The regulation also would require safeguards to prevent unlawful access to the agents. It is important to note that congress stated in PL 104-132 that the regulation had to be designed to provide for the appropriate availability of BSATs for legitimate purposes such as research and education (Public Law 104-132, 1996). Access to BSATs was to be restricted for those with no legitimate reason while, at the same time, maintaining availability to those with a legitimate reason. The new regulations were incorporated into Part 72 of Title 42 of the Code of Federal Regulations (C.F.R. Part 72) by which the CDC regulated the interstate shipment of etiologic agents. The final regulation (42 C.F.R. Parts 72.6, 72.7, and Appendix A [the Select Agent list]) became effective April 15, 1997 and was in effect until February 7, 2003.

The regulatory climate changed after September 11, 2001 and the subsequent dissemination of *Bacillus anthracis* spores through the U.S. mail that resulted in 22 cases of anthrax and 5 deaths (Cole, 2003). During the ensuing investigation, the FBI contacted CDC’s Select Agent Program to request a list of all laboratories possessing *B. anthracis*, in particular those that possessed the Ames strain, which was the strain isolated and identified in all of the human anthrax cases as well as environmental samples resulting from the attack (Keim et al., 2011). However, the list was probably incomplete as only those laboratories that had transferred or received the agent since April 1997 had been required to register with the CDC Select Agent Program. This fact highlighted, from a law enforcement perspective, a loophole in the Select Agent Regulation that needed to be corrected through legislation.

Controls to access select agents were strengthened after the anthrax mailing incident. On October 26, 2001, Public Law 107-56 entitled “Uniting and Strengthening America by Providing Appropriate Tools Required to Intercept and Obstruct Terrorism (USA PATRIOT) Act was signed by President George W. Bush. The USA PATRIOT Act affected who could handle or possess BSATs. Section 817 of the USA PATRIOT Act amended Chapter 10 (Biological Weapons) of Title 18, United Sates Code (U.S.C.). The first change incorporated as subparagraph (b) in Section 275 [18 U.S.C. Part 175(b)] stated: “Whoever knowingly possesses any biological agent, toxin, or delivery system of a type or in a quantity that, under the circumstances, is not reasonably justified by a prophylactic, protective, bona fide research, or other peaceful purpose, shall be fined under this title, imprisoned not more than 10 years, or both.” In the context of subparagraph 175(b), the terms “biological agent” and “toxin” did not include a biological agent or toxin in its naturally occurring environment as long as the biological agent or toxin had not been cultivated, collected, or otherwise extracted from its natural source (Public Law 107-56, 2001). Some examples that might have been problematic without this understanding are the presence of a plague-infected rodent on one’s property or the extraction of castor oil from castor bean seeds with the resulting residue (i.e., mash) containing the toxin ricin. The second statement in a new section 175b restricted who could possess BSATs. It stated that “No restricted person described in subsection (b) shall ship or transport in interstate or foreign commerce, or possess in or affecting commerce, any biological agent or toxin, or receive any biological agent or toxin that has been shipped or transported in interstate or foreign commerce, if the biological agent or toxin is listed as a Select Agent (Public Law 107-56, 2001).” The categories of restricted persons delineated in Section 175b are listed in Table 1 and the current Select Agent List is shown in Table 2.

**Table 1:** **Categories of restricted persons as described in the USA PATRIOT Act of 2001^a^**.

A person who is under indictment for a crime punishable by imprisonment for a term exceeding 1 year.
A person who has been convicted in any court of a crime punishable for a term exceeding 1 year.
A person who is a fugitive from justice.
A person who is an unlawful user of any controlled substance (as defined in section 102 of the Controlled Substances Act (21 U.S.C. 802).
An alien illegally or unlawfully in the United States.
A person who has been adjudicated as a mental defective or has been committed to any mental institution.
An alien (other than one lawfully admitted for permanent residence) who is a national of a country as to which the Secretary of State has made a determination (that remains in effect) that such country has repeatedly provided support for acts of international terrorism.
A person who has been discharged from the Armed Services of the United States under dishonorable conditions.

*^a^Abstracted from Public Law 107-56 (2001)*.

**Table 2:** **HHS and USDA select agents and toxins^a^**.

**HHS select agents and toxins**
Abrin
Botulinum neurotoxins^b^
Botulinum neurotoxin producing species of *Clostridium*^b^
Conotoxins (short, paralytic alpha conotoxins containing the following amino acid sequence: X_1_CCX_2_PACGX_3_X_4_X_5_X_6_CX_7_)^c^
*Coxxiella burnetti*
Crimean-Congo hemorrhagic fever virus
Diacetoxyscirpenol
Eastern Equine Encephalitis virus^d^
Ebola virus^b^
*Francisella tularensis*^b^
Lassa fever virus
Lujo virus
Marburg virus^b^
Monkeypox virus^d^
Reconstructed replication competent forms of the 1918 pandemic influenza virus containing any portion of the coding regions of all eight gene segments (Reconstructed 1918 influenza virus)
Ricin
*Rickettsia prowazekii*
SARS-associated coronavirus (SARS-CoV)
Saxitoxin
South American Hemorrhagic Fever viruses (Chapare, Guanarito, Junin, Machupo, Sabia)
Staphylococcal enterotoxins A, B, C, D, E subtypes
T-2 toxin
Tetrodotoxin
Tick-borne encephalitis complex (flavi) viruses (Far Eastern subtype, Siberian subtype)
Kyasanur Forest disease virus
Omsk hemorrhagic fever virus
Variola major virus (Smallpox virus)^b^
Variola minor virus (Alastrim)^b^
*Yersinia pestis*^b^
**Overlap select agents and toxins**
*Bacillus anthracis*^b^
*Bacillus anthracis* Pasteur strain
*Brucella abortus*
*Brucella melitensis*
*Brucella suis*
*Burkholderia mallei*^b^
*Burkholderia pseudomallei*^b^
Hendra virus
Nipah virus
Rift Valley fever virus
Venezuelan equine encephalitis virus^d^
**USDA select agents and toxins**
African horse sickness virus
African swine fever virus
Avian influenza virus^d^
Classical swine fever virus
Foot-and-mouth disease virus^b^
Goat pox virus
Lumpy skin disease virus
*Mycoplasma capricolum*^d^
*Mycoplasma mycoides*^d^
Newcastle disease virus^d^^,^^e^
Peste des petite ruminants virus
Rinderpest virus^b^
Sheep pox virus
Swine vesicular disease virus
**USDA plant protection and quarantine select agents and toxins**
*Peronosclerospora philippinensis* (*Peronosclerospora sacchari*)
*Phoma glycinicola* (formerly *Pyrenochaeta glycines*)
*Ralstonia solanacearum*
*Rathayibacter toxicus*
*Sclerophthora rayssiae*
*Synchytrium endobioticum*
*Xanthomonas oryzae*

*^a^Information obtained at: http://www.selectagents.gov/SelectAgentsandToxinsList.html*.

*^b^Denotes Tier 1 agent*.

*^c^C, cysteine residues are all present as disulfides, with the 1st and 3rd cysteine, and the 2nd and 4th cysteine forming specific disulfide bridges; The consensus sequence includes known toxins α-MI and α-GI (shown above), as well as α-GlA, Ac1.1a. α-CnlB; X1, any amino acid(s) or Des-X; X2, asparagine of histidine; P, proline; A, alanine; G, glycine; X3, arginine or lysine; X4, asparagine, histidine, lysine, arginine, tyrosine, phenylalanine, or tryptophan; X5, tyrosine, phenylalanine, or tryptophan; X6, serine, threonine, glutamate, aspartate, glutamine, or asparagine; X7, any amino acid(s) or Des-X and; “Des-X”, an amino acid does not have to be present at this position. For example, if a peptide sequence were XCCHPA, then the related peptide CCHPA would be designated as Des-X*.

*^d^Select agents that meet any of the following criteria are excluded from the requirements of this part: any low pathogenic strain of avian influenza virus, South American genotype of eastern equine encephalitis virus, west African clade of Monkey pox virus, any strain of Newcastle disease virus, which does not meet the criteria for virulent Newcastle disease virus, all subspecies *Mycoplasma capricolum* except subspecies *capripneumoniae* (contagious caprine pleuropneumonia), all subspecies *Mycoplasma mycoides* except subspecies *mycoides* small colony (Mmm SC) (contagious bovine pleuropneumonia), any subtypes of Venezuelan equine encephalitis virus except for subtypes lAB or lC, and Vesicular stomatitis virus (exotic); Indiana subtypes VSV-lN2, VSV-lN3, provided that the individual or entity can verify that the agent is within the exclusion category*.

*^e^A virulent Newcastle disease virus (avian paramyxovirus serotype 2) has an intracerebral pathogenicity index in day-old chicks (*Gallus gallus*) of 0.7 or greater or has an amino acid sequence at the fusion (F) protein cleavage site that is consistent with virulent strains of Newcastle disease virus. A failure to detect a cleavage site that is consistent with virulent strains does not confirm the absence of a virulent strain*.

Another law that would impact the regulation of BSATs, the “Public Health Security and Bioterrorism Preparedness and Response Act of 2002” (PL 107-188), was signed by President Bush on June 12, 2002. Title II of this Act directed the HHS Secretary to: “(1) establish and maintain (and review at least biennially) a list of BSATs that have the potential to pose a severe threat to public health and safety; (2) provide for the regulation of transfers of listed agents and toxins; (3) provide for the establishment and enforcement of standards and procedures governing the possession and use of listed BSATs; (4) require registration with the HHS Secretary of the possession, use, and transfer of listed BSATs; and (5) provide appropriate safeguards and security requirements for persons possessing, using, or transferring a listed agent or toxin commensurate with the risk such agent or toxin poses to public health and safety (Public Law 107-188, 2002).” It also authorized the HHS Secretary to inspect the facilities of persons subject to the above requirements to ensure their compliance with the regulations.

In addition, the HHS Secretary, in consultation with the Attorney General and now with the Secretary of Homeland Security as well, established safeguards and security requirements for persons wishing to posses, use, or transfer a BSAT. These requirements dealt with limiting access to BSATs by ensuring that registered persons: “(1) provide access to only those individuals whom the registered person determines has a legitimate need to handle or use BSATs; (2) provide names and other identifying information (e.g., fingerprints) to both the HHS Secretary and Attorney General for those individuals identified as needing access; (3) deny access to BSATs by individuals whom the Attorney General has identified as “restricted persons” as defined in the USA PATRIOT Act (see Table 1); and (4) limit or deny access to BSATs to an individual who is reasonably suspected by any Federal law enforcement or intelligence agency of (a) committing a crime set forth in section 2332b(g)(5) of Title 18, U.S.C.; (b) knowing involvement with an organization that engages in domestic or international terrorism or with any other organization that engages in intentional crimes of violence, or (c) being an agent of a foreign power (as defined in section 1801 of Title 50, U.S.C.) (Public Law 107-188, 2002). The results of the Attorney General’s security risk assessment (SRA) are provided to the HHS Secretary (specifically to the Director of CDC’s Division of Select Agents and Toxins) where the determination as to whether the individual is to be granted or denied access to BSATs is made. The revised select agent regulations are found in 42 C.F.R. Part 73 (Select Agent Regulation, , 42 C.F.R. Part 73). At the end of 2012, there were over 400 registered facilities and more than 13,171 individuals (including scientists, technicians, and various support personnel) approved for access to select agents.

Another major change is found in Subtitle B (Agricultural Bioterrorism Protection Act of 2002) of PL 107-188, which directs the Secretary of the USDA to establish and maintain a list of BSATs that he/she determines has the potential to pose a severe threat to animal or plant health, or to animal or plant products. The criteria for inclusion are described in Public Law 107-188 (2002). For the animal pathogens they include: (1) availability and effectiveness of pharmacotherapy and prophylaxis to treat and prevent any illness; (2) economic impact; inclusion on the Office International des Epizooties (OIE) A and B lists; and (3) presence on the Australia Group List (2014). For plant pathogens, they include: (1) the effect of exposure to the agent on plant health and on the production and marketability of plant products; (2) the ability to detect the agent and diagnose the infection during its early stages; (3) whether the agent was non-native or exotic; and (4) the economic importance of the host plant. The non-biological criteria of economic consequences and effect on international trade agreements were of paramount importance when considering agents for inclusion on the USDA BSAT list (Table 2) (Pimental et al., 2000). Thus, these agents are designated Select Agents not because they necessarily pose a threat to animal health but because they pose a threat to national security (National Research Council, 2010). This is in contrast to the HHS list where the impact on public health and safety was a primary factor for inclusion. The USDA promulgated two select agent regulations: 9 C.F.R. Part 121, which governs BSATs that have the potential to pose a severe threat to animal health, or to animal products (Select Agent Regulation, 9 C.F.R. Part 121); and 7 C.F.R. Part 331, which governs BSATs that pose a severe threat to plant health or plant products (Select Agent Regulation, 7 C.F.R. Part 331). The three select agent regulations are virtually identical with respect to requirements and how they are structured. BSATs that are on both the HHS and USDA Select Agent lists are called “overlap agents and toxins” and are jointly regulated by both HHS and USDA (see Table 2).

Shortly after the passage of the Public Law 107-188, CDC’s Division of Select Agents and Toxins and USDA’s Animal and Plant Health Inspection Service (APHIS) jointly established the Federal Select Agent Program (FSAP) to coordinate the regulation of BSATs. The FSAP is responsible for carrying out the provisions of the USA PATRIOT Act and the Public Health Security and Bioterrorism Preparedness Act through three select agent regulations (42 CFR Part 73, 7 CFR Part 331, and 9 CFR Part 121). FSAP promotes laboratory safety and security through regulations, registering laboratories, conducting onsite inspections, and providing guidance to the regulated community. FSAP also approves and tracks shipments of BSATs, investigates reports of their theft, loss, or release, collects information on BSATs identified by diagnostic laboratories, and provides select agent facility status information to federal decision makers during responses to natural and intentional disasters (Blaine, 2012).

Some government agencies working with BSATs have developed and implemented additional, more stringent safety, security, and agent accountability programs for their laboratories (The President, 2009; Shurtleff et al., 2012), sometimes in response to adverse press coverage. For example, findings during the Amerithrax investigation (United States Department of Justice, 2010; Willman, 2011) led the U.S. Department of Defense (DOD) to issue a security directive entitled “Safeguarding Biological Select Agents and Toxins” (Directive 5210.88) for military laboratories (Carr et al., 2004; Pastel et al., 2006) working with BSATs (United States Department of Defense, 2004). In accordance with Directive 5210.88, the U.S. Army developed and implemented Army Regulation 50-1 (Department of the Army, 2008), a comprehensive biosurety program for all Department of the Army laboratories working with BSATs, including those at USAMRIID, Ft. Detrick, Frederick, MD, USA, which were at the center of the Amerithrax investigation.

## Select Agent List

The Select Agent List is considered by some to be primarily an instrument of biosecurity although it can be argued that the Select Agent Regulation(s) also reduce biosafety concerns through the regulation of laboratories working with BSATs (Casadevall and Relman, 2010).

The initial list of BSATs, which was part of the original regulation in 1997, contained 42 agents and toxins including some agents that could infect both human beings and animals (e.g., *B. anthracis* and *Y. pestis*), but did not include those affecting only animals or plants. The original BSATs were selected (i.e., Select Agents) from the Australia Group List of Human and Animal Pathogens and Toxins for Export Control (Australia Group List, 2014) with input from experts from inside and outside the government. Considerations for selection by the committee included prior weaponization, effect on human health, infectious dose, degree of contagiousness, route(s) of infection, and the availability of effective medical countermeasures and vaccines. There was extensive discussion with ASM as well as input from other scientists during the comment period The Australia Group List currently consists of 59 agents that can infect both human beings and animals (37 viruses, 20 bacteria, and 2 fungi) and 19 toxins (Australia Group List, 2014).

The select agent regulations require biennial review of the list and agents can be added or removed based on new information or better understanding. For example, the reconstructed 1918 influenza virus was added in 2005 and SARS-associated coronavirus (SARS-CoV) was added in 2013. Information provided by both the science and security communities is evaluated during the review process. The current BSAT list consists of 65 agents and toxins of which 34 are HHS BSATs, 10 are overlap BSATs, 14 are USDA BSATs, and 7 are USDA Plant Protection and Quarantine BSATs (Table 2).

The current BSATs differ significantly in their pathogenicity and ability to be utilized as an agent of bioterrorism, and therefore, the risk that they might pose to human being, animal, and plant health and safety varies significantly (National Science Advisory Board for Biosecurity, 2009). However, the 2005 Select Agent Final Rule applied the same regulatory controls regardless of the agent. Casadevall and Relman and the NSABB have noted that this broad application of the regulations has made it difficult to conduct legitimate research using less pathogenic BSATs (National Science Advisory Board for Biosecurity, 2009, Casadevall and Relman, 2010). Therefore, the NSABB in a 2009 report recommended that the list of BSATs be reduced or stratified (National Science Advisory Board for Biosecurity, 2009). Subsequently, Executive Order 13546, which was signed on July 2, 2010, directed the Secretaries of HHS and Agriculture to designate those BSATs that pose the greatest risk of deliberate misuse with the greatest potential for mass casualties or devastating effects to the economy, critical infrastructure, or public confidence as Tier 1 agents and toxins (see Table 2) and amend their respective regulations to establish security standards specific to Tier 1 agents and toxins (The President, 2010). A federal panel using a number of criteria including information from the intelligence community selected 13 agents deemed to be Tier 1 (Bhattacharjee, 2011).

The finding that the *B. anthracis* spores used in the 2001 anthrax letters presumably came from a federal laboratory facility (United States Department of Justice, 2010) played an important role in designing the security changes to address the insider threat. Specific changes in the regulations related to Tier 1 agents are described in Table 3. The NSABB also issued a number of recommendations related to hiring and employment practices and to fostering an awareness of biosecurity and promoting responsible conduct as an approach to reducing the insider threat (National Science Advisory Board for Biosecurity, 2011). Some laboratories developed and implemented personnel reliability programs to address the insider threat prior to the amendment of the regulations (Higgins et al., 2013).

**Table 3:** **Select agent regulation (42 CFR Part 73, 7 CFR Part 331, and 9 CFR Part 121) requirements for working with Tier 1 BSATs^a^**.

Section	Requirement
15(b)	Entities with Tier 1 select agents and toxins must conduct annual insider threat awareness briefings on how to identify and report suspicious behaviors (training).
12(d)	The biosafety plan must include an occupational health program for individuals with access to Tier 1 agents and toxins, and those individuals must be enrolled in the occupational health program (occupational health).
14(b)	Entities with Tier 1 agents must provide the following additional Information in the incident response plan: (i) A plan for how the Entity will respond to the activation of the alarm system or Information on an intruder in the laboratory; (ii) Procedures on how the entity will notify the appropriate federal, state, or local law enforcement agencies of suspicious activity that may be criminal in nature and related to the entity, its personnel, or its select agents or toxins (incident response plan).
11(f)(2)	Entities must describe procedures for how an entity’s Responsible Official (RO) will coordinate their efforts with the entity’s safety and security professionals to ensure security of Tier 1 select agents and toxins and share appropriate, relevant information, which may affect the security plan (security plan).
11(f)(4)(iv)	A requirement for three barriers (physical structure that is designed to prevent access to Tier 1 agents by unauthorized persons) (security plan).
11(f)(4)(v)	A requirement for intrusion detection system (security plan).
11(f)(4)(viii)	Entity must determine the response time for response force (security plan).
11(f)(4)(vii)	Entity must describe procedures to ensure that security is maintained in the event of the failure of the access control system due to power disruption (security plan).
11(f)(1);11(f)(3)	Persons with access to Tier 1 BSATs must have additional pre-access suitability and on-going assessment requirements (security plan).
11(f)(4)(ii)	Limit access to laboratories and storage facilities outside of normal business hours to only those specifically approved by RO or designee(s) (security plan).
11(f)(4)(iii)	Procedures must be in place for screening visitors, their property, and vehicles at the entry and exit points to the areas registered for Tier 1 BSATs (security plan).

*^a^Specific guidance for these and other requirements of the Select Agent Regulations can be found in the following documents: Guidance for Meeting the Training Requirement of the Select Agent Regulations, October 1, 2012 edition; Occupational Health Program Guidance Document for Working with Tier 1 Select Agents and Toxins, October 1, 2012 edition; Incidence Response Plan Guidance Document, September 4, 2014 edition; and, Security Guidance for Select Agent or Toxin Facilities, July 5, 2013 edition, which can be found at http://www.selectagents.gov*.

## Impact of the Select Agent Regulations

Determining the positive or negative impact of these regulations in preventing terrorists or criminals from accessing dangerous pathogens has challenges. Difficulties in linking information with impact, availability of data, and verification of anecdotal statements are among the reasons. An examination of the theft, loss, and release incident reports (APHIS/CDC Form 3) submitted to APHIS or CDC between 2004 and 2010 revealed no reports dealing with BSAT theft (Henkel et al., 2012). Among the 727 reports received, 88 (12%) were loss reports and 639 (88%) were release reports. The final disposition of the 88 loss reports indicated that there was one confirmed loss of a BSAT (*Coccidioides immitis*) during shipment (out of 3412 BSAT transfers conducted during that time). An investigation of this incident by the FBI concluded that it was destroyed during processing at a commercial shipping facility in the U.S. (Henkel et al., 2012). One positive impact of the regulations may be that there is currently a monitoring program in place that can help to foster sustained security in U.S. labs.

The regulations were “intended to provide a potential benefit to society by both restricting access to certain microorganisms and toxins and creating a legal infrastructure for the prosecution of individuals who are found to be in possession of these organisms and toxins without proper registration” (Casadevall and Relman, 2010). These authors further propose that these regulations “mitigate risk by imposing a strict regulatory environment on laboratories working with select agents” (Casadevall and Relman, 2010). They agree that the regulations make an important contribution to biosecurity for those microorganisms that no longer circulate in the environment (e.g., variola virus) and for those that, until recently, were considered difficult to isolate from natural sources (e.g., Ebola virus). However, they and others [e.g., Ostfield (2009)] correctly note that many of the select agents are endemic to the U.S. (and elsewhere) and can be isolated from natural sources by individuals with some microbiological training. Thus, the regulation may be of limited benefit in cases where the select agent can be isolated from the natural environment and perhaps provide a false sense of security. This was one of the main arguments used by proponents for the stratification of the Select Agent List according to the agents’ potential use as a biothreat agent, with regulatory requirements and procedures calibrated against this stratification (National Research Council, 2010).

To date, many of the proposed negative impacts of these regulations are based on anecdotal evidence. There is, however, some work to document any unintentional consequences of the regulatory policies. Casadevall and Relman (2010) suggested that many microbial collections were destroyed by their curators when these regulations came into effect in the U.S. rather than register. To circumvent the loss of biodiversity, the Select Agent Program worked hard to find “homes” for any culture collection that entities no longer wanted to retain as a result of the new regulation. However, additional data are needed to determine the impact of the regulations on microbial collections. For example, it is not clear how many microbial collections were lost prior to these regulations because they were not funded in a sustainable manner. It is also possible that it was not the regulations themselves that caused destruction but the increased cost of maintenance under regulatory constraints.

Casadevall and Relman (2010) also suggested that these regulations could or have impaired work on BSATs. In this regard, over 20% of select agent researchers surveyed in 2004 and 2005 noted that the regulations were affecting their ability to collaborate domestically and internationally, and about 40% claimed that they had to use research funding to make required security upgrades (Sandia National Laboratories, 2006). Shurtleff et al. (2012) suggested that the regulations (i.e., both the Select Agent and Army regulations) impeded work in BSL-4 laboratories by adding extra requirements. A 2006 Stimson Center Study found that the main complaints of select agent researchers were monetary, the time and costs of security upgrades and procedures, bureaucratic time sinks, the tedium of inventorying samples, and barriers to international collaboration (Fischer, 2006). Nevertheless in a recent survey, an overwhelming number of investigators (93.4%) responded that these agents should be regulated and that the SRA was the most effective component of the regulation (Sutton, 2009). On the other hand, many respondents were worried that inadvertently violating these regulations would have a negative impact on their careers and potentially thwart the goals of the regulations.

Dias et al. (2010) conducted a bibliometric analysis of the *B. anthracis* and Ebola virus archival literature to determine whether there were negative consequences of the underlying legislation on research with these agents. After correcting for papers that would not be subject to these regulations (e.g., review articles), the number of annual peer reviewed publications increased markedly after the regulation went into effect. The amount of research funding also increased post-September 11 (Dias et al., 2010). However, the cost per publication, using funding amounts identified in both the NIH CRISP database and the RAND Corporation’s RaDIUS database, also increased. Before 2002, the average number of *B. anthracis* research papers per million dollars of funding was 17. After 2002, the average number was only 3 papers per million dollars. For Ebola virus, before 2002 the average number of papers per million dollars was 14, which subsequently fell to 6 papers per million dollars. Research papers on a non-regulated control organism (*Klebsiella pneumoniae*) declined from 26 to 17 per million dollars of funding. While the authors recognize the softness of their data, they claim that there has been a two- to fivefold increase in doing select agent research. However, this increase cannot be attributed solely to the costs associated with the Select Agent regulations as this study did not consider the nature of the research being conducted, the increased cost of purchasing small laboratory animals and primates, and of conducting animal studies at BSL-3 and -4. Moreover, there has been a trend over the past few years of academic institutions requiring investigators to put an increasing proportion of their salaries on research grants. Based on the above findings, it is clearly apparent that further analysis is needed to accurately determine the financial impact.

In the wake of the passage of the underlying legislation of the Select Agent regulations, more than one high-profile scientist announced publicly that they would abandon select agent research rather than fulfill the legal requirements (Gaudioso and Salerno, 2004). It is possible that some scientists who have been working with these agents prior to the regulations bristled at the changes brought by the regulations while those who entered the field after the regulations went into effect have been more accepting. Controlling for funding, Dias et al. (2010) actually detected an influx of new scientists that entered “live-pathogen” select agent research after the laws were passed, but many did not stay (these may be graduate students who changed fields after completing their degree). An influx of new scientists was not observed among control organism researchers. However, a pattern of decline was observed in international collaboration on *B. anthracis* but not Ebola virus research. Overall, Dias et al. (2010) found that select agent research became less centralized (i.e., involved more institutions) after the laws were enacted and military institutions became more collaborative in “live-pathogen” *B. anthracis* and Ebola virus research.

In phone interviews conducted to determine whether their findings were consistent with individual experiences, Dias et al. (2010) found that research partnerships were not affected by the regulations, and that most of the respondents perceived increased collaboration and diversity of expertise within the field after 2002. However, they pointed out that the collaborative process was made significantly slower and more tedious due to the restrictions placed on organism transfer (particularly with foreign partners) and laboratory access. Unfortunately, Dias et al. (2010) did not ascertain whether difficulty in obtaining an Export License (both in the U.S. and abroad) for BSAT materials was a significant factor but they did note that nearly all respondents complained of the increased paperwork they were legally obligated to fill out.

## International Approaches

There have been a number of international efforts to promote biosafety and biosecurity due to the threat of bioterrorism and emerging infectious diseases. The 2004 edition of the World Health Organization’s (WHO) *Laboratory Biosafety Manual* contains, for the first time, a discussion of biosecurity (World Health Organization, 2004). Microorganisms are classified by risk group (Table 4). The risk groups correspond to the biosafety level required to safely work with the microorganism (e.g., Risk Group 3 requires biosafety level 3 containment and practices). Laboratory biosecurity is viewed as a complement to laboratory biosafety in that while they mitigate different risks, they share the common goal of keeping biological agents safe and secure in the areas where they are used and stored (World Health Organization, 2006).

**Table 4:** **Classification of infective microorganisms by risk group^a^**.

Hazard group	Definition	Examples
1	An organism that is most unlikely to cause human disease	Non-mammalian associated microorganisms
2	An organism that may cause human disease and which might be a hazard to laboratory workers but is unlikely to spread in the community	*Legionella* spp., *Clostridium botulinum, Staphylococcus aureus*, Herpes simplex, Influenza virus
3	An organism that may cause severe human disease and presents a serious hazard to laboratory workers	*Bacillus anthracis, Brucella* spp., *Mycobacterium tuberculosis, Salmonella typhi, Shigella dysenteriae*
4	An organism that causes severe human disease and is a serious hazard to laboratory workers. It may present a high risk or spread to the community and there is usually no effective prophylaxis or treatment	Variola, Ebola, Marburg, Lassa, Machupo, Junin

*^a^Modified from World Health Organization (2004) and Hoffman (1994)*.

Because most countries have never had to respond to a bioterrorism incident, the level of public awareness and concern about this issue is likely to be considerably less than in the U.S. While many countries (e.g., Germany) experienced anthrax hoaxes in 2001, they were quickly discovered to be false alarms. Germany did not respond to the events of 2001 by introducing new biosecurity legislation as was done in the U.S. (Tucker, 2007). Tucker (2007) points out that while the U.S. framed bioterrorism prevention as a security issue and responded by tightening controls on a targeted list of BSATs that could be used as weapons, Germany (and a number of other countries) viewed the threat of bioterrorism mainly in public health terms, as a subset of the broader challenge of infectious diseases (Tucker, 2007). So while the U.S. Select Agent Regulation focuses primarily on pathogens that may be used by a bioterrorist, Germany relies on a framework of biosafety laws and regulations, dating back to 1900, which are designed to ensure the safe handling of dangerous pathogens by legitimate researchers and to minimize the risks to public health and the environment from legitimate research activities. Among the German laws related to biosafety are The Reich Epidemic Act of 1900 requiring scientists wishing to work with dangerous pathogens to meet certain educational requirements and to be licensed by the state; the Genetic Engineering Act of 1993; the Plant Protection Act of 1998; the Regulation on Health and Safety at Work Act of 1996; the Regulation on Health and Safety Related to Activities Involving Biological Agents of 1999; the Infection Protection Act of 2000; and the Animal Infectious Disease Act of 2001. These and other laws and regulations governing biosafety were developed incrementally over more than a century. Germany currently regulates personnel and/or facilities for all of the agents designated as Hazard Group 3 or 4 and require the equivalent of a security clearance for those working with Hazard Group 4 agents (National Research Council, 2009). Because of this approach, the total number of microbial and toxin agents covered by German biosafety regulations is larger than the U.S. Select Agent List (Tucker, 2007). Other countries using a similar approach are Canada and Switzerland (National Research Council, 2009). Some countries regulate via lists of “select agents,” which vary in length, composition, and requirements. Examples of such lists are Australia (22 agents); South Korea (32 agents); France (37 agents); Japan (51 agents), and the United Kingdom (U.K.) (82 agents) (National Research Council, 2009).

In the aftermath of the September 11 attacks in the U.S., the U.K. passed the Antiterrorism, Crime and Security Act of 2001 (http://www.hmso.gov.uk/acts/acts2001/20010024.htm), which shifted the focus from laboratory biosafety to biosecurity. Part VII tightens controls on access to 82 dangerous pathogens and toxins that are specified in Schedule 5 of the Act, establishes the power to vet personnel working in laboratories with these agents and mandates security requirements (National Research Council, 2003). Schedule 5 currently lists 41 viruses, 4 rickettsiae, 21 bacteria, 14 toxins, and 2 fungi that are subject to this Act.

There have also been increased multinational efforts to improve biosafety and biosecurity in order to prevent or deter the use of biological agents as weapons and to achieve global health security. These efforts are centered on the implementation of international instruments for both non-proliferation [e.g., Biological Weapons Convention (BWC) and United Nations Security Council Resolution 1540 (UNSCR 1540)] and public health [e.g., WHO International Health Regulations (IHR)]. A full discussion of these agreements is beyond the scope of this paper and the interested reader is referred to Bakanidze et al. (2010) for additional information. All 192 UN Member States are required to meet the core capacity requirements of the IHR, which entered into force in 2007 (International Health Regulations, 2005). “Core Capacity 8,” the laboratory core capacity, emphasizes the concept that building laboratory capacity to support a public health system requires a strong focus on biosafety and biosecurity. The BWC, which has 170 States Parties, entered into force in 1975 (http://www.unog.ch/bwc). The BWC States Parties hold Review Conferences every 5 years in an effort to enhance compliance. At the 2008 BWC meeting of States Parties, it was stated that “biosafety and biosecurity measures contribute to preventing the development, acquisition, or use of biological and toxin weapons and are appropriate means of implementing the BWC” and also that “pursuing biosafety and biosecurity measures could also contribute to the fulfillment of other respective international obligations and agreements, such as the revised IHR of the WHO.” UNSCR 1540 was adopted unanimously on April 28, 2004 and established for the first time legally binding obligations on all UN Member States to “develop and enforce effective measures against the proliferation of weapons of mass destruction (WMD),” which includes biological weapons (UN Security Council, 2004). UNSCR 1540 also recognizes that non-state proliferation is a threat to the peace under the terms of Chapter VII of the UN Charter and requires every UN Member State to criminalize various forms of non-state actor involvement in WMD and its related activities in its domestic legislation and to enforce such legislation. An *ad hoc* committee (known as the 1540 Committee) was involved in the implementation of this resolution. As part of its mission, it developed matrices to be used as tools to help UN Member States implement the resolution. The matrix for biological weapons and related materials identified the areas where domestic controls should be implemented and enforced (Table 5).

**Table 5:** **Areas where domestic controls should be implemented and enforced in order to prevent the proliferation of biological weapons under UN Security Council Resolution 1540^a^**.

Measures to account for secure production
Measures to account for secure use
Measures to account for secure storage
Measures to account for secure transport
Regulations for physical protection of facilities/materials/transports
Licensing/registration of facilities/persons handling biological materials
Reliability check of personnel
Measures to account for/secure/physically protect means of delivery
Regulations for genetic engineering work
Other legislation/regulations related to safety and security for biological materials

*^a^Adapted from Bakanidze et al. (2010)*.

## Conclusion and Outlook

Security and defense against biological threats, whether natural or intentional will most likely continue to be a high priority for the foreseeable future (Ryan and Glarum, 2008). Carus compiled data suggesting that there is an increased interest in biological agents by criminals and terrorists (Carus, 2002). He identified four methods used by criminals and terrorists to acquire biological agents, either: (1) purchase them from legitimate suppliers; (2) steal them; (3) produce them; or (4) use material of natural origin contaminated with biological agents (Carus, 2002). Purchase from a legitimate supplier and theft accounted for more than half of known instances. However, bioterrorism is a multifaceted problem requiring a multifaceted solution. In response to the threat and actual use of a biological agent by a terrorist, the U.S. passed legislation and promulgated regulations (i.e., Select Agent Regulations) to restrict access to pathogens that pose a risk to human beings, animals, plants, or animal and plant products to those with a legitimate need who have been approved to receive, ship, or possess a BSAT. The regulations also require the theft, loss, or release of one of the BSATs to be reported to the Select Agent Program. Background checks and personnel reliability programs were instituted to reduce the insider threat. Other countries have addressed these issues through biosafety regulations and laws. The IHR and UNSCR 1540 has fostered international awareness of the global threat of bioterrorism and emphasized the necessity for member states to strengthen the biosecurity and biosafety of their laboratories.

In the literature, there is discussion that the current regulations may have the unintended consequences of impeding legitimate research by imposing additional requirements, slowing international collaborations and increasing the cost of research. Some scientists and policy experts (Ostfield, 2009; Casadevall and Relman, 2010) have noted that these regulations will not stop someone with microbiological skills from isolating a BSAT from nature, but do provide a legal framework for prosecuting someone who possesses a BSAT illegally. To facilitate legitimate BSAT research that does not require highly virulent organisms, the select agent regulations established a procedure by which attenuated or avirulent strains that do not pose a severe threat to public health and safety, animal health, or animal products may be excluded from the requirements of the select agent regulations. Exclusion is based upon consultations with subject matter experts and a review of relevant published studies and unpublished data provided by the entity requesting the exclusion. The exclusion of BSATs has been occurring for more than a decade. Excluded bacteria and viruses are shown in Table 6. The availability of excluded strains has facilitated research on many of these agents.

**Table 6:** **Excluded bacterial and viral select agents^a^**.

Agent	Strain	Effective date
Avian influenza virus	Recombinant vaccine reference strains of the H5N1 and H5N3 subtypes	05-07-2004
*Bacillus anthracis*	Strains devoid of pX01 and pX02	02-27-2003
*Bacillus anthracis*	Strains devoid of pX02 (Sterne)	02-27-2003
*Brucella abortus*	Vaccine strain (Δ*norD*Δ*znuA*)	06-02-2011
*Brucella abortus*	Vaccine strain S2308Δ*pgm*	08-09-2006
*Brucella abortus*	Vaccine strain 19	06-12-2003
*Brucella abortus*	Vaccine strain RB51	05-07-2003
*Burkholderia pseudomallei*	Bp82 (a Δ*purM* mutant of strain 1026b)	04-14-2010
*Burkholderia pseudomallei*	B0011 (a Δ*asd* mutant of strain 1026b)	12-07-2011
*Coxiella burnetii*	Nine Mile Strain Phase II, plaque purified clone 4	10-15-2003
Eastern equine encephalitis virus	South American genotypes	12-04-2012
Ebola virus	ΔVP30 replication incompetent virus	01-02-2013
*Francisella tularensis* subsp*. novicida (F. novicida)*	Utah 112 (ATCC 15482)	02-27-2003
*Francisella tularensis subsp. novicida (F. novicida)*	All strains	10-24-2014
*Francisella novicida-*like strains	All strains	10-24-2014
*Francisella tularensis* subsp. *holarctica*	LVS (live vaccine strain; includes NDBR 101 lots, TSI-GSD lots, and ATCC 29684)	02-27-2003
*Francisella tularensis* subsp. *tularensis*	B-38 (ATCC 6223)	02-27-2003
Junin virus	Vaccine strain Candid No. 1	02-07-2003
Lassa fever virus	Mopeia/Lassa arenavirus construct ML-29	03-02-2005
Monkeypox virus	West African clade of Monkeypox virus	12-04-2012
Rift Valley fever virus	Vaccine strain MP-12	02-07-2003
Rift Valley fever virus	Vaccine candidate strain ΔNSs-ΔNSm-ZH501	03-12-2012
SARS-Coronavirus	NATtrol™ treated SARS-CoV molecular controls	02-08-2013
Venezuelan equine encephalitis virus (VEE)	Subtypes ID and IE	12-04-2012
VEE	Vaccine candidate strain V3526	05-05-2003
VEE	Vaccine strain TC-83	02-07-2003
*Yersinia pestis*	Pgm^−^(Δpgm) e.g., EV or various substrains such as EV 76	03-14-2003
*Yersinia pestis*	Lcr^−^(e.g., Tjiwidej S, CDC A1122)	02-27-2003

*^a^Descriptions of the excluded agents can be found at http://www.selectagents.gov/Exclusions-usda.html, http://www.selectagents.gov/exclusions-hhs.html, and http://www.selectagents.gov/exclusions-overlap.html. Excluded toxins (not listed here) can be found at the latter two websites*.

The development of codes of conduct and the education of scientists about dual-use research of concern are additional efforts by the scientific community that may strengthen biosecurity without the development of additional laws and regulations. The life sciences community previously set a precedent for self-governance in the way it addressed potential safety and environmental dangers in the then-fledgling field of genetic engineering. A conference in Asilomar, California in 1975 (Berg et al., 1975) brought recombinant DNA science and technology to the public’s attention and led to the development of guidelines by a new National Institutes of Health Committee, the Recombinant DNA Advisory Committee, that governs all recombinant DNA work at institutions that receive any federal funds for that purpose. The recombinant DNA guidelines addressed how to conduct experiments using this technology safely and forbid the performance of certain types of experiments. The flexibility afforded by guidelines means that they can be updated as new scientific judgments on risk are made. The use of these guidelines has expanded beyond institutions that receive government funds. By being proactive, scientists avoided restrictive legislation.

Many policy makers and scientists believe that the development, documentation, and reinforcement of norms regarding the beneficial aspects of science as well as identifying those activities that are fundamentally intolerable are best done by scientists (National Security Council, 2009). The *National Strategy for Countering Biological Threats* (National Security Council, 2009) seeks to facilitate a “culture of responsibility” through engaging the global life sciences community, encouraging: (1) sustained dialog on the development of behavioral norms; (2) the development of a code of ethics; (3) the development of training materials; and (4) efforts to explore community-based approaches for identifying and addressing irresponsible conduct. Codes of conduct for the global sciences community are widespread. Many apply to the individual (e.g., professional oaths and codes of ethics) and workplace (e.g., recombinant DNA guidelines). Most are voluntary, have minimal penalties and/or no mechanism for ensuring compliance, and primarily rely on the ethical behavior of the individual. The scientific community is best served by a balanced approach between voluntary codes of conduct and regulations/laws that protect society from the misuse of BSATs, while not hindering legitimate biological research.

Life science knowledge and technologies continues to advance at a rapid pace, which presents challenges to regulations (e.g., Select Agent Regulations) that govern pathogen security. The total synthesis of a viral genome and recovery of infectious viruses (Cello et al., 2002), enhancing the virulence of a minimally virulent non-select agent virus (Rosengard et al., 2002), and the creation of a bacterial cell controlled by a chemically synthesized genome (Gibson et al., 2010) are examples of technological advances that could be used to circumvent the regulations. The ability to keep up with these scientific advances will be a challenge that will require both regulatory and non-regulatory approaches (National Research Council, 2003).

## Conflict of Interest Statement

The author declares that the research was conducted in the absence of any commercial or financial relationships that could be construed as a potential conflict of interest. To ensure the robustness and objectivity of our peer review process, the Specilty Chief Editor of the journal, Dr. Kenneth Berns, was consulted and approved the acceptance of the manuscript for publication.
